# Online consultations in mental healthcare: Modelling determinants of use and experience based on an international survey study at the onset of the pandemic

**DOI:** 10.1016/j.invent.2022.100571

**Published:** 2022-09-05

**Authors:** Tom Van Daele, Kim Mathiasen, Per Carlbring, Sylvie Bernaerts, Agostino Brugnera, Angelo Compare, Aranzazu Duque, Jonas Eimontas, David Gosar, Lise Haddouk, Maria Karekla, Pia Larsen, Gianluca Lo Coco, Tine Nordgreen, João Salgado, Andreas R. Schwerdtfeger, Eva Van Assche, Sam Willems, Nele A.J. De Witte

**Affiliations:** a Thomas More University of Applied Sciences, Belgium; b Department of Clinical Medicine, University of Southern Denmark, Denmark; c Centre for Digital Psychiatry, Mental Health Services of Southern Denmark, Denmark; d Department of Psychology, Stockholm University, Stockholm, Sweden; e Department of Human & Social Sciences - University of Bergamo, Bergamo, Italy; f Universidad Internacional de Valencia, Valencia, Spain; g Cibersalud, Mallorca, Spain; h Department of Clinical Psychology, Institute of Psychology, Vilnius University, Vilnius, Lithuania; i Department of Child, Adolescent & Developmental Neurology, University Children's Hospital, University Medical Centre Ljubljana, Slovenia; j Department of Psychology, University of Rouen, France; k Department of Psychology, University of Cyprus, Cyprus; l Mental Health Services in the Region of Southern Denmark, Vejle, Denmark; m Department of Psychology, Educational Science and Human Movement- University of Palermo, Italy; n Haukeland University Hospital, University of Bergen, Bergen, Norway; o University of Maia, Portugal & Center of Psychology at University of Porto, Portugal; p Institute of Psychology, University of Graz, Graz, Austria

**Keywords:** Digital mental health, COVID-19, Online consultations, Telepresence

## Abstract

**Introduction:**

While online consultations have shown promise to be a means for the effective delivery of high-quality mental healthcare and the first implementations of these digital therapeutic contacts go back nearly two decades, uptake has remained limited over the years. The onset of the COVID-19 pandemic dramatically altered this relative standstill and created a unique turning point, with a massive amount of both professionals and clients having first hands-on experiences with technology in mental healthcare.

**Objective:**

The current study aimed to document the uptake of online consultations and explore if specific characteristics of mental health professionals across and beyond Europe could predict this.

**Methods:**

An international survey was designed to assess mental health professionals' (initial) experiences with online consultations at the onset of the pandemic: their willingness to make use of them and their prior and current experiences, alongside several personal characteristics. Logistic mixed-effects models were used to identify predictors of the use of online consultations, personal experience with this modality, and the sense of telepresence.

**Results:**

A total of 9115 healthcare professionals from 73 countries participated of which about two-thirds used online consultations during the initial COVID-19 outbreak. The current study identifies multiple determinants relating to the use and experience of online consultations, including the professionals' age, experience with the technology before the outbreak, the professional context, and training.

**Conclusions:**

Despite strong evidence supporting the relevance of training in digital mental health, this is clearly still lacking. Nevertheless, the COVID-19 pandemic presented a first, and potentially transformative, experience with online consultations for many healthcare professionals. The insights from this study can help support professionals and, importantly, (mental) healthcare organisations to create optimal circumstances for selective and high-quality continued use of online consultations.

## Highlights

• The Covid-19 pandemic increased uptake of online consultations in mental health.
• This quantitative study included 9115 mental health professionals from 73 countries.
• Several determinants, including training, use of only consultations including training
• Investing in training might create better circumstances for online consultations.

## 1 Introduction

Online consultations have been a part of mental healthcare for close to two decades (Andersson and Carlbring, 2021). Online consultations can be defined as digital contacts related to psychological counseling or psychotherapy which clients and mental health professionals communicate via text, audio, video, or a combination of all these (De Witte et al., 2021). Digital therapeutic contacts have consistently been found to be a means for the effective delivery of high-quality mental healthcare (Ebert et al., 2018), for one-on-one virtual contact (Berryhill et al., 2019a; Berryhill et al., 2019b; Ruwaard et al., 2012) and for group interventions (Banbury et al., 2018). However, the underlying dynamics of such online contacts remain inconclusive, as well as whether these contacts can also occur and take shape in a virtual environment (de Bitencourt Machado et al., 2016; Miloff et al., 2020). Nevertheless, online consultations do seem to allow for similar dynamics between clients and mental health professionals as compared to conventional therapy (Haddouk et al., 2018).

Although the practice of online consultations has been hinted at as a potential ‘growth area’ (Martin et al., 2020) and supported through guidelines from professional associations (e.g., Joint Task Force for the Development of Telepsychology Guidelines for Psychologists from the American Psychological Association, 2013; Ordre des Psychologues du Québec, 2013), uptake has remained limited over the years. Online consultations were only used on a regular basis by a limited number of highly specialized practitioners or sporadically on an ad hoc, limited basis by most: only when circumstances required so or simply not at all (Gaebel et al., 2021). A wide variety of factors play a role in the successful uptake of technology in mental healthcare, including the expected amount of effort required, the added value of technology and the broader social and organizational context of mental health professionals (Titov et al., 2018). The Unified Theory of Acceptance and Use of Technology (UTAUT) states that each of these can in turn be impacted by individual mental health professionals' characteristics, such as gender, age, and experience, although these moderators haven't always been identified in relation to the acceptance of digital health interventions (Venkatesh et al., 2003; Philippi et al., 2021). Taken together, the successful dissemination and implementation of technology in mental healthcare remains a challenge, despite an increasingly expanding field of research (Van Daele et al., 2021; Prieto-Fidalgo et al., 2021).

The onset of the COVID-19 pandemic dramatically altered this relative standstill and created a unique turning point, with a massive amount of both professionals and clients having first hands-on experiences with technology in mental healthcare (Wind et al., 2020). A large survey with licensed U.S. psychologists on their telepsychology use before and during the COVID-19 pandemic showed that only 7 % of treatment was provided using telepsychology before the pandemic, whereas up to 85.5 % of their clinical work was performed online during the pandemic (Pierce et al., 2021). Online video consultations were the technological modality most widely adopted by the overall majority of professionals around the world, from Asia (Liu et al., 2020), over Europe (Van Daele et al., 2020) to the US (Gentry et al., 2021). Even in countries that previously had already invested substantially in e-mental health services, such as Australia, a surge in uptake was noticed (Marshall et al., 2020).

As the need for online consultations soared during the first COVID-19 wave and resulting lockdowns across the world, little was known, however, on how mental health professionals dealt with this. More specifically, it was unclear to what extent mental health professionals were eager to make use of the technology, how skilled they were, to what extent they felt comfortable and if and how they managed to make use of an often novel way of getting in touch with their clients. There is initial evidence that therapists struggled with implementing common therapeutic skills (i.e., therapeutic communication and relational abilities such as warmth or empathy) in online settings in comparison to in-person settings (Lin et al., 2021). Several national and international initiatives moved forward to provide mental healthcare professionals with basic support to facilitate uptake and to increase their overall comfort with the use of technology in ‘the new normal’ (Karayianni et al., 2022). One of these ad hoc initiatives was an international survey aimed at gaining insights into these changing dynamics, which was initiated by the Project Group on eHealth of the European Federation of Psychologists' Associations (EFPA; https://www.efpa.eu/working-groups/ehealth). A qualitative analysis made clear that the overall majority of therapists had received limited to no training concerning optimal use of technology in mental healthcare, and accordingly had several concerns regarding the implementation of online consultations as a part of clinical practice (De Witte et al., 2021).

The quantitative data analysis of this international survey, which is the focus of the current paper, primarily aimed to document the uptake of online consultations and explore if specific characteristics of mental health professionals across and beyond Europe could predict this. Furthermore, we also aimed to obtain additional insights into the overall experience of mental health professionals relying on online consultations: to what extent did the professionals feel comfortable making use of online consultations and what was their perceived level of telepresence? Telepresence is a common concept in the domain of online consultations and concerns the impression of being physically present, which can be experienced to a varying extent by both mental health professionals and patients, and is known to impact the strength of the therapeutic relationship (Haddouk et al., 2018). Overall, as the survey was a rapid response to a sudden, global change in clinical practice, the aim of this article is to provide a detailed and documented overview of what – in hindsight – may be a unique event: a potential turning point in technology-enhanced delivery of mental healthcare.

## 2 Material and methods

### 2.1 Design

The study was designed as a cross-sectional survey of recent use of online consultations as well as present characteristics of the psychologists and their context, including additional retrospective questions on use of online consultations prior to the COVID-19 outbreak. The study was approved by the ethical committee of the Department of Applied Psychology at Thomas More University of Applied Sciences (ID 1920_16). All participants provided informed consent.

### 2.2 Recruitment

An international survey was created and distributed by EFPA's Project Group on eHealth in March 2020. From March 18 through May 5, 2020, mailing lists, social media, and other distribution channels of EFPA and related organisations were used to disseminate the survey as widely as possible, resulting in inclusion of participants from 73 countries worldwide. The full list of participating countries can be found in Appendix A. A total of 9115 mental healthcare professionals took part in the study. Completion of all questions from the survey was not required. All given responses were included in the current descriptive statistics and analyses.

### 2.3 Survey

The survey was designed to assess mental health professionals' (initial) experiences with online consultations at the onset of the pandemic: their willingness to make use of them, their prior and current experiences, and their concerns, alongside several personal characteristics. The survey was made available in 17 languages through Qualtrics online survey software, with translations being provided by researchers and professionals in the field of psychology with knowledge of local and national contexts. Participants generally completed the questionnaire in about 4 to 10 min. This article focuses on the questions concerning the use of online consultations, the experience with this modality and the level of telepresence in relation to individual characteristics of the mental health professional.

### 2.4 Statistical analyses

Descriptive statistics and summary graphs, were made using the free software environment R (R Core Team, 2021) supplemented with R package lmerTest for the hierarchical statistical modelling (Kuznetsova et al., 2017). The statistical software Stata was used for both primary and secondary analyses (StataCorp, 2021). Hierarchical statistical modelling was preceded by visual inspection of the data. The visual inspection phase aimed to give a first overview of the data. No statistical analyses were implemented in this phase since only a model, taking into account all relevant factors, is meaningful when analyzing such large datasets in which comparisons have a high likelihood of reaching statistical significance.

#### 2.4.1 Primary analysis

The primary analysis was conducted using a logistic mixed-effects model with restricted maximum likelihood estimator. Use of online consultations in recent days was used as a dichotomous response variable with the response categories ‘yes’ and ‘no’ (the questionnaire also offered the response category ‘no, but I intend to’ which, due to a small size and being a negative response, was included in the ‘no’ category). The following potential predictors were considered in univariate models and in a mutually adjusted model: gender, age, length of professional experience, main professional situation (context) and prior training in the use of online consultations. As random effects, individuals were nested within countries and intercepts were allowed to vary. All available data were included. Assumptions on linearity of the continuous variables ‘age’ and ‘length of professional experience’ were assessed by visual inspection of deviance residual plots. Colinearity was assessed using variance inflation factor (VIF). Model specification was assessed by visual inspection of observed and model predicted outcomes.

#### 2.4.2 Secondary analyses

For the secondary analyses we applied linear multilevel mixed effects models. Since outcomes could only assume one of five values, and due to moderate deviations of the normality assumptions of the residuals, the models were fitted using robust standard errors. The overall experience of use of online consultations and the sense of telepresence, respectively, were response variables in two different multivariate linear models considering the potential predictors: the continuous variable ‘length of professional experience’ and the categorical variables ‘previous experience using online consultations’ and ‘prior training’. Once again, individuals were nested within countries and intercept was allowed to vary. All available data were included.

Both models assumed normally distributed and homoscedastic error terms at individual level and at country level, as well as linearity of continuous predictors. These assumptions were examined by visual inspection of q-q normality plots and residual plots at both individual level and country level. Since outcomes could only assume one of five values, and due to moderate deviations of the normality assumptions of the residuals, the models were fitted using robust standard errors.

## 3 Results

The characteristics of the sample can be seen in Table 1. The majority of participants were psychologists. The ‘Other’ category contains self-specified professions, such as mental health nurse, psychotherapist, or university professor, but was often used to describe a setting (e.g., school, private practice) rather than a profession. Many participants have extensive professional experience, but very few have received any form of training in digital mental health.

**Table 1:** Characteristics of the sample.

Sample characteristics	n	%
Profession		
Psychologist	6611	72.5
Psychiatrist	42	0.5
Other	1345	14.8
Previous training on online consultations		
Yes	832	9.1
No	8256	90.6
Main professional situation		
Self employed	4862	53.3
Group practice	159	1.7
Health care organisation	843	9.2
Mental health care organisation (not further specified)	1001	11.0
Other	1123	12.3
Gender		
Male	1264	13.9
Female	6714	73.7
Non-binary	21	0.2
Age (in years)		
<30	803	8.8
30 to 39	2672	29.3
40 to 49	2405	26.4
50 to 59	1306	14.3
60 to 69	685	7.5
70 and above	110	1.2
Professional experience (in years)		
<10	3099	34.0
10 to 19	2572	28.2
20 to 29	1322	14.5
30 to 39	687	7.5
40 and above	220	2.4

*Note.* To provide a conservative estimate of the percentages, percentages are relative to the full sample (i.e., N = 9115) and take into account (varying numbers of) missing data for each of the sample characteristics.

### 3.1 General overview

Recent use of online consultations, the overall experience of using online consultations and the experienced level of telepresence during online consultations are presented graphically. The variables are further presented according to prior experience with the use of online consultations, previous training, the professional context of the participants, gender, age and length of professional experience of the participants.

#### 3.1.1 Recent use of online consultations

**Fig. 1:**
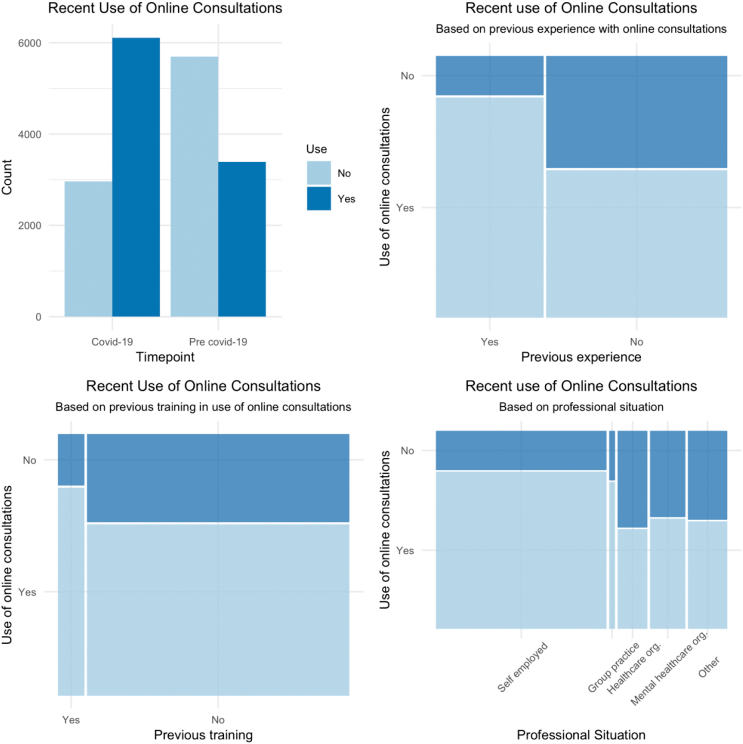
Visual representation of participants' recent use of online consultations in the context of the COVID-19 pandemic.

Two thirds (n = 6105, 67.3 %) replied that they had used online consultations in recent days. This contrasts with the finding that just over one third (n = 3389, 37.3 %) of respondents had experience in the use of online consultations prior to the outbreak of COVID-19. Visual inspection of the data shows that use in recent days was higher among psychologists with previous experience using these delivery formats and/or having received training in their use (Fig. 1). Use in recent days varied between participants practicing in different professional situations, with more frequent use among self-employed psychologists. The use of online consultations did not seem to vary according to gender and, interestingly, neither according to age or length of professional experience (not shown here).

#### 3.1.2 Overall experience using online consultations

**Fig. 2:**
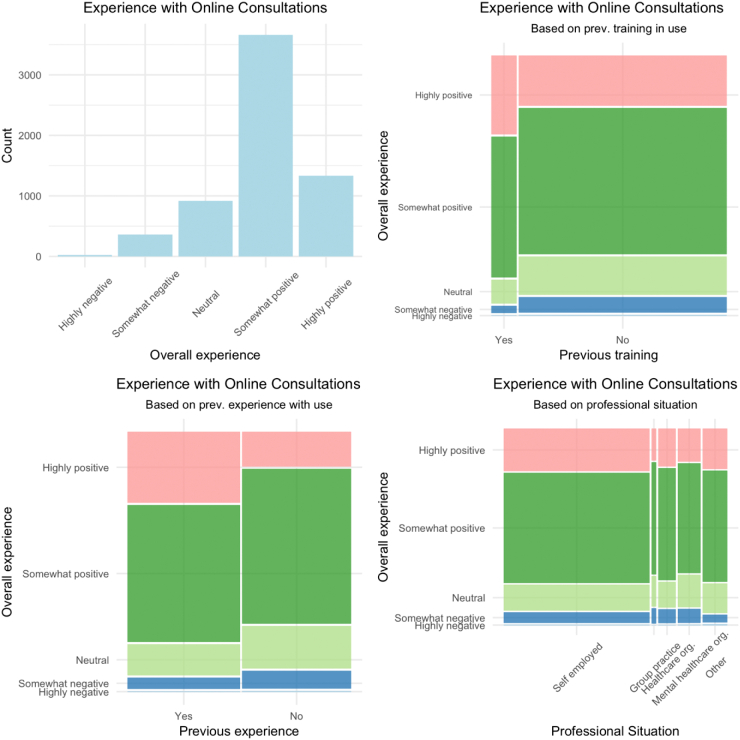
Visual representation of participants' experiences with online consultations in the context of the COVID-19 pandemic.

The majority of the respondents had a ‘somewhat positive’ (n = 3663) or ‘highly positive’ (n = 1334) experience using online consultations (Fig. 2). A larger proportion of positive scores was seen among those with previous experience and/or prior training in the usage of online consultations. Visual inspection suggests only very slight differences in level of overall experience based on the professional context of the participant. Gender, age and length of professional experience did not seem to influence the overall experience of using online consultations.

#### 3.1.3 Level of telepresence

**Fig. 3:**
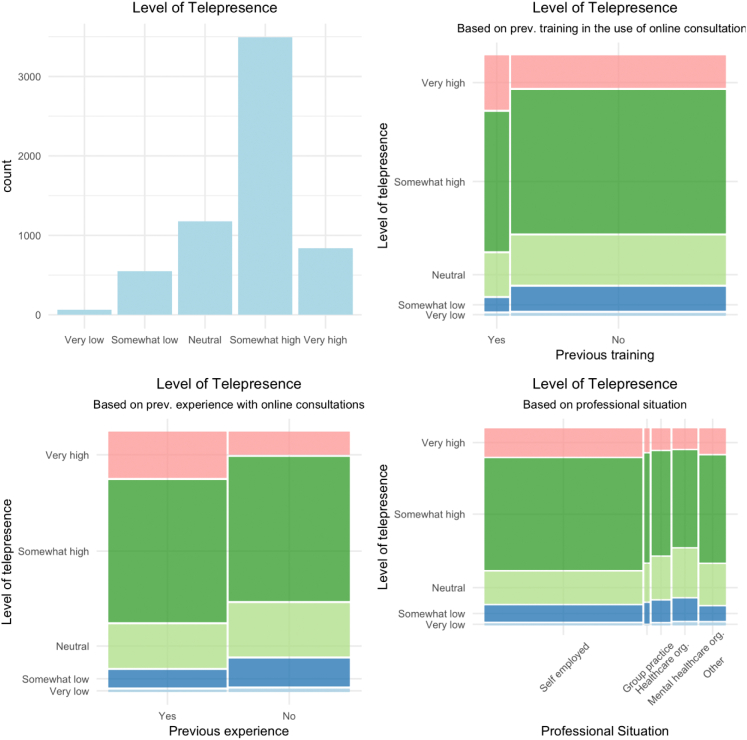
Visual representation of participants' level of telepresence.

Most participants experienced a ‘somewhat high’ (n = 3491) or ‘very high’ (n = 839) level of telepresence, with almost 30 % (n = 1788) nevertheless reporting neutral to (very) low levels (Fig. 3). More positive responses were seen among those who had prior training and/or previous experience using online consultations. Again, gender, age and length of professional experience did not seem to influence the level of telepresence.

### 3.2 Primary analysis: predictors of use of online consultations at the initial COVID-19 outbreak

Results on the use of online consultations during the COVID-19 outbreak can be seen in Table 2. We found no colinearity between predictor (all VIF's < 10). There was no indication of misspecification of the model. There were no significant effects of gender. Age showed a small, yet significant, effect with an adjusted odds ratio slightly lower than one (0.98), indicating that the higher the age the lower the odds of having used online consultations during the outbreak. The opposite was found for length of professional experience. Results indicated that longer professional experience increased the odds of online consultations use. Moreover, those with no previous training and/or no experience using online consultations prior to the pandemic had significantly lower odds of engaging in the use of online consultations during the outbreak. Furthermore, the professional situation influenced use of online consultations; we found that, compared to being self-employed, those working in healthcare or mental healthcare organisations (and in ‘other’ professional contexts) were less likely to have used online consultations recently.

**Table 2:** Primary analysis of the predictors of the use of online consultations at the COVID-19 outbreak (N = 7632).

*Predictors*	*Categories*	Unadj.	Adjusted^a^		
		*Odds ratios*	*Odds ratios*	*95* *% CI*	*p*
Gender	Male	Ref.			
	Female	0.93	1.09	0.93–1.28	0.274
	Non-binary (X)	1.16	1.24	0.35–4.33	0.733
Age (in years)		1.01	0.98	0.97–0.99	<0.001***
Professional experience (in years)		1.01	1.03	1.02–1.04	<0.001***
Previous training	No	Ref.			
	Yes	2.17	1.80	1.45–2.39	<0.001***
Previous experience with online consultations	No	Ref.			
	Yes	5.42	4.66	4.06–5.35	<0.001***
Professional situation	Self-employed	Ref.			
	Group practice	0.79	1.01	0.67–1.54	0.952
	Healthcare organisation	0.28	0.31	0.26–0.38	<0.001***
	Mental healthcare organisation	0.33	0.37	0.32–0.45	<0.001***
	Other	0.35	0.40	0.34–0.48	<0.001***

a Mutually adjusted, *p < .05, **p < .01, ***p < .001.

### 3.3 Secondary analyses: predictors of experiences and telepresence when using online consultations

As can be seen in Table 3, a small but significant effect of professional experience in years was seen on the overall experience of using online consultations. Not having previous experience using online consultations or no prior training in the use was significantly negatively associated with the overall experience.

**Table 3:** Experience using online consultations (N = 5756).

*Predictors*	Categories	Unadj.	Adjusted^a^		
			*Coefficient, B*	*95* *% CI*	*p*
Professional experience (in years)		0.01	0.00	0.00–0.01	<0.001***
Previous experience with online consultations	No	Ref.			
	Yes	0.26	0.24	0.22–0.26	<0.001***
Prior training	No	Ref.			
	Yes	0.24	0.20	0.14–0.26	0.002**

a Mutually adjusted *p < .05, **p < .01, ***p < .001.

A similar pattern was observed when analyzing the sense of telepresence. Longer professional experience was associated with a slight positive effect on the sense of telepresence, while lack of prior experience and training significantly reduced the experience of telepresence. The results can be seen in Table 4.

**Table 4:** Secondary analysis: Experience of telepresence.

Telepresence when using online consultations (N = 5651)					
*Predictors*	Categories	Unadj.	Adjusted^a^		
			Coefficient, B	*95* *% CI*	*p*
Professional experience (in years)		0.01	0.01	0.00–0.01	<0.001***
Previous experience with online consultations	No	Ref.			
	Yes	0.24	0.22	0.18–0.25	<0.001***
Prior training	No	Ref.			
	Yes	0.20	0.18	0.11–0.24	<0.001***

a Mutually adjusted *p < .05, **p < .01, ***p < .001.

## 4 Discussion

Implementation of online consultations has been limited during the last two decades despite a longstanding and annually growing research tradition and established effectiveness across settings and target groups. The surge in the use of online consultations due to the initial COVID-19 outbreak has allowed us to document and model the determinants of use and experience of online consultations in a large international sample. Therefore, the current study aimed to record the uptake of online consultations at the COVID-19 outbreak and explore if any characteristics of mental health professionals could predict use of online consultations. Furthermore, we also aimed to explore the overall experience and perceived level of telepresence of mental health professionals relying on online consultations.

Two-thirds of the large international (albeit mostly European) professional sample used online consultations at the COVID-19 outbreak. In line with the findings from Pierce et al. (2021) in the U.S., current findings show that the pandemic led to a first experience with online consultations for most mental healthcare professionals around the world. Since the circumstances required swift adoption of technology, implementation processes are likely to have been suboptimal. Nevertheless, results show that experiences with online consultations were mostly positive.

The current study corroborates previous work in relation to the relevance of professionals' age, training and experience with the technology for the uptake of eHealth (e.g., Mair et al., 2012; Sinclair et al., 2013; Venkatesh et al., 2003). Experience with online consultations prior to the COVID-19 outbreak and training in this technological modality were the strongest predictors of use, experience, and experienced level of telepresence. Nevertheless, the scarcity of training was clearly identified as well. Despite international telepsychology guidelines identifying supervision and training as key practice domains (McCord et al., 2020) and the current study implementing a very liberal interpretation of training (a brief workshop could be enough, see also De Witte et al., 2021), training rates were low and unsatisfactory. In line with this, Hames et al. (2020) identify many challenges experienced by psychology training programs when forced to rapidly change from in-person services to telehealth services in response to COVID-19. Furthermore, Perry et al. (2020) found that an important barrier to using telepsychology was therapists' lack of self-efficacy due in part to insufficient opportunities for training.

Additionally, results showed that individuals having a longer professional career in the field of mental health were more inclined to use online consultations during the first COVID-19 outbreak, while also reporting higher levels of telepresence and a more positive overall experience. In contrast, a more advanced age in itself reduced the likelihood of using online consultations, which is in line with previous work and can be related to lower technology literacy (Sinclair et al., 2013). The current findings show that if you hold everything else constant, then the odds of using online consultations lowered slightly with age. If, however, you hold age (and everything else) constant, then the more experienced psychologists are to some extent more likely to use online consultations. Nevertheless, this specific finding pertaining to the role of professional experience is novel and the effects of age and professional experience were small, so they should be interpreted tentatively and replicated in further research. In contrast with existing theory, gender did not significantly influence the use of online consultations (Venkatesh et al., 2003). However, this is in line with a recent UTAUT model validation study in the context of in somatic and mental healthcare, which was not able to find a moderating effect of gender on the intention to use internet- and mobile-based interventions (Philippi et al., 2021). While Philippi et al. (2021) also did not observe a significant effect of age and experience on intention to use technology, the current study complements these findings by suggesting that age and experience could be relevant factors for the actual uptake of online consultations in mental healthcare.

Next to these individual factors, the professional situation was also found to be important, as being active in (mental) healthcare organisations reduced the likelihood of using online consultations. Since the questionnaire was completed at the beginning of the first outbreak of COVID-19 on the European continent, it cannot be determined whether this was merely a delay (due to implementation barriers that might exist in larger organisations and different legal regulations across countries) or whether this represents a more fundamental reluctance toward this technological modality in such settings. The systematic review of Mair et al. (2012) also supports the relevance of contextual integration of eHealth systems (e.g., administrative support, policy support, and standards) and organisation size has also been identified as potential barrier (Kruse et al., 2018). Examples of large mental health organisations rapidly transferring their operations online exist, however the transition remains technically and organisationally challenging, even in organisations with experience in the matter (Chen et al., 2020).

### 4.1 Strengths and limitations

The current study was conducted at an interesting and specific turning point when a large number of mental healthcare professionals were suddenly exposed to a challenging situation which required them to change their current practices. Previous research has shown that predictors and moderators of acceptance and use of technology can very between settings and contexts. We aimed to document the change process and investigate predictors of the use of online consultations in a large and diverse international sample of healthcare professionals. This implies that, although the variance caused by differences between countries was included in the mixed effects model, the study was not designed to inform on regional differences between countries (of which some contributed only a few individuals). While the pandemic resulted in quick and similar implementation processes of online consultations across countries, also facilitated by relaxed national regulations and guidelines promoting adoption (De Witte et al., 2021), future studies should look into differences between countries in the long-term integration of digital interventions in mental healthcare. Adoption rates of online consultations might also have already fluctuated during the span of the pandemic. Another limitation that should be addressed in future research is that the current study is a cross-sectional, correlational study, which precludes certainty regarding causal directions.

### 4.2 Conclusions and recommendations

While the COVID-19 outbreak presented a first experience with online consultations for many healthcare professionals, it might have been a transformative one since the findings show that having used online consultations in the past can promote further implementation. However, sufficient training on online consultations and e-mental health implementation should also be provided to support professionals and, importantly, (mental) healthcare organisations to create optimal circumstances for selective and high-quality continued use of online consultations We hope our appeal not only sensitizes professionals in the mental health field to the issue but also induces key decision and policy makers to adopt training on online consultations as an important part of the wider re-envision of post-COVID mental healthcare.

## Declaration of interests

The authors declare that they have no known competing financial interests or personal relationships that could have appeared to influence the work reported in this paper.

## Appendix A

Albania, Andorra, Argentina, Armenia, Australia, Austria, Belgium, Bhutan, Bosnia and Herzegovina, Brazil, Bulgaria, Canada, Colombia, Croatia, Cyprus, Czech Republic, Democratic Republic of the Congo, Denmark, Estonia, Finland, France, Gabon, Germany, Greece, Guyana, Hong Kong (S.A.R), Hungary, Iceland, India, Indonesia, Ireland, Israel, Italy, Jamaica, Japan, Jordan, Kazakhstan, Kiribati, Kuwait, Latvia, Lebanon, Lithuania, Luxemburg, Malta, Mauritius, Mexico, Morocco, Netherlands, New Zealand, Norway, Peru, Philippines, Poland, Portugal, Qatar, Romania, Russian Federation, Rwanda, San Marino, Serbia, Slovakia, Slovenia, South Africa, Spain, Sweden, Switzerland, Thailand, The former Yugoslav Republic of Macedonia, Turkey, Ukraine, United Arab Emirates, United Kingdom of Great Britain and Northern Ireland, United States of America.
